# The Impact of Compassion from Others and Self-compassion on Psychological Distress, Flourishing, and Meaning in Life Among University Students

**DOI:** 10.1007/s12671-022-01891-x

**Published:** 2022-04-29

**Authors:** Kevin Ka Shing Chan, John Chi-Kin Lee, Eric Kwan Wai Yu, Arita W. Y. Chan, Angel Nga Man Leung, Rebecca Y. M. Cheung, Chin Wa Li, Raymond Ho-Man Kong, Junjun Chen, Sarah Lai Yin Wan, Christine Hau Yu Tang, Yen Na Yum, Da Jiang, Lixun Wang, Chun Yip Tse

**Affiliations:** 1 Department of Psychology, The Education University of Hong Kong, Tai Po, Hong Kong; 2 Centre for Psychosocial Health, The Education University of Hong Kong, Tai Po, Hong Kong; 3 Department of Curriculum and Instruction, The Education University of Hong Kong, Tai Po, Hong Kong; 4 Centre for Religious and Spirituality Education, The Education University of Hong Kong, Tai Po, Hong Kong; 5 Department of Literature and Cultural Studies, The Education University of Hong Kong, Tai Po, Hong Kong; 6 Department of Early Childhood Education, The Education University of Hong Kong, Tai Po, Hong Kong; 7 Centre for Child and Family Science, The Education University of Hong Kong, Tai Po, Hong Kong; 8 Department of International Education, The Education University of Hong Kong, Tai Po, Hong Kong; 9 Department of Education Policy and Leadership, The Education University of Hong Kong, Tai Po, Hong Kong; 10 Department of Special Education and Counselling, The Education University of Hong Kong, Tai Po, Hong Kong; 11 Integrated Centre for Wellbeing, The Education University of Hong Kong, Tai Po, Hong Kong; 12 Department of Linguistics and Modern Language Studies, The Education University of Hong Kong, Tai Po, Hong Kong; 13 Centre for Language in Education, The Education University of Hong Kong, Tai Po, Hong Kong

**Keywords:** Compassion from others, Self-compassion, Resilience, Psychological distress, Flourishing, Meaning in life

## Abstract

**Objectives:**

Research shows that compassion from others and from the self may enable university students to face, overcome, and bounce back from adversity and generate a greater sense of thriving and meaning in life. However, the underlying processes are largely unknown. The present study aimed to examine the associations of compassion with psychological distress, flourishing, and meaning in life among university students and explore the mechanisms underlying these associations.

**Methods:**

A total of 536 Hong Kong university students completed questionnaires measuring their experiences of compassion from others, self-compassion, resilience, psychological distress, flourishing, and meaning in life.

**Results:**

Serial mediation analyses showed that compassion from others was associated positively with self-compassion, which was, in turn, linked to greater resilience and consequently lower levels of psychological distress and higher levels of flourishing and meaning in life.

**Conclusions:**

Our findings reveal the associations of compassion from others and self-compassion with the well-being and life meaning of university students. The findings highlight the importance of being open and receptive to love and kindness from others. The findings also point to the importance of developing a caring attitude toward oneself.

Life education and positive education, linked with students’ personal and values development, have gained increasing importance in school and university education (Lee et al., 2021). While university education provides students with opportunities for personal growth and values clarification, university life can be challenging and stressful (Fong & Loi, 2016). The challenges and stressors faced by university students may include handling heavy academic loads, living independently away from home, making new friends, paying tuition fees, and developing post-graduation plans (Crocker & Luhtanen, 2003; Hurst et al., 2013). If students perceive their life demands as exceeding their coping resources, they may feel inadequate and incompetent and engage themselves in negative self-evaluation, self-criticism, and self-shame, which can increase their risks of depression, anxiety, substance use, and suicide (Beiter et al., 2015; Levine et al., 2020; Pedrelli et al., 2015; Zhao et al., 2020).

Research shows that university students have higher rates of mental health difficulties compared to the general population (Steptoe et al., 2007). For instance, a systematic review has found the prevalence rate of depressive symptoms in this group to be 30.6%, which was considerably higher than that in the general population (9%) (Ibrahim et al., 2013). Given this high prevalence of mental health difficulties among university students, it is of paramount importance to help them build resilience against their stressors. One potential way to develop stress resilience is through the provision of compassionate social support and the enhancement of compassionate self-care (Neff & McGehee, 2010; Ozbay et al., 2007).

Compassion refers to being sensitive to suffering, with a desire to relieve and avert it (Dalai Gilbert & Choden, 2013; Jinpa, 2015; Lama, 1995; Ricard, 2015). Being compassionate involves an awareness of suffering, an empathetic concern about it, a hope to witness its alleviation, and an eagerness to help reduce it (Strauss et al., 2016). According to Gilbert (2014), there can be different orientations and flows of compassion that involve the applications of compassionate capacities. Specifically, an individual may extend compassion to others and to the self and receive compassion from the self and from others (Gilbert, 2014). It is worth noting that these different flows of compassion can influence one another (Kirby et al., 2019). For instance, a person who receives compassion from others may also become more self-compassionate and give greater compassion to others (Kirby et al., 2019).

Compassion from others refers to care, kindness, and support from other people during difficult times (Gilbert et al., 2017). It is related to the extent to which individuals are surrounded by supportive and caring social environments in which people show compassionate intentions and behaviors (e.g., being soothing, listening, and accepting) (Hermanto et al., 2016). In order to accept and experience compassion from others fully, individuals have to increase their openness and receptiveness to it and reduce their fear of and resistance to receiving it (Gilbert et al., 2011).

According to the social mentality theory (Gilbert, 1989, 2009), receiving compassion from others (e.g., through secure attachment relationships) may facilitate the development of the soothing system in emotion regulation, which can enable one to calm and comfort the self in difficult times. Some studies have corroborated these views by showing that compassion may have dual positive effects on physiological and psychological well-being (Di Bello et al., 2020; Stellar et al., 2015). Specifically, compassion may suppress sympathetic activity and enhance parasympathetic influence, which can calm potential stress reactions (Stellar et al., 2015). Moreover, compassion may increase vagally mediated heart rate variability, which is related to higher positive affect and lower negative affect (Di Bello et al., 2020).

An individual who continuously receives compassion from others may increasingly internalize compassion for the self, resulting in higher levels of self-soothing capacities (Gilbert, 1989, 2009). Notably, there is growing evidence that receiving compassion from others (i.e., compassion flowing from others to self) may facilitate the enhancement of self-compassion (i.e., compassion flowing from self to self) (Kirby et al., 2019). In particular, individuals who engage in compassionate social relationships are likely to have higher levels of self-acceptance, self-kindness, and self-warmth (Hermanto & Zuroff, 2016; Hermanto et al., 2016).

Self-compassion is a self-caring attitude when confronting adversity or perceived inadequacy (Neff, 2003). From a social psychological perspective (Neff, 2003), self-compassion comprises three elements: self-kindness (versus self-judgment), common humanity (versus isolation), and mindfulness (versus over-identification). In particular, self-kindness entails the tendency to be warm and accepting, instead of harsh and critical, toward the self when encountering hardship. Common humanity involves the understanding that difficulties, imperfections, and failures are ordinary, and not unusual, in people’s life. Mindfulness refers to the nonjudgmental awareness of moment-by-moment experiences, without over-identifying or trivializing negative ones.

Self-compassion can be a psychological resource for coping with daily hassles and life obstacles (Allen & Leary, 2010; Dvořáková et al., 2019). When facing difficult or stressful times, individuals who adopt a self-compassionate stance may be better able to extend kindness, warmth, and affirmation to themselves for regaining emotional equanimity (Ewert et al., 2021). By adopting nonjudgmental attitudes toward their adversities and normalizing their suffering through viewing it as a universal human experience, these individuals may have greater resilience to get through, bounce back, and move on from negative experiences (Bluth et al., 2018).

Compassion can facilitate individuals to adapt to and recover from life adversities, and ultimately, this may empower them to reconstrue unpleasant events as benign, meaningful, or growth promoting (Ferreira et al., 2021; Wong & Yeung, 2017). Such positive reappraisals of negative experiences may help transform affective distress to positive affect and generate a durable sense of thriving and meaning in life (Pérez-Aranda et al., 2021; Yela et al., 2020). The potential psychological outcomes are lower levels of negative affect, languishing mood, and distress as well as higher levels of subjective happiness, life satisfaction, and flourishing (Brenner et al., 2018; Cavalcanti et al., in press; MacBeth & Gumley, 2012; Marsh et al., 2018; Matos et al., 2017; Satici et al., 2013).

Some studies have shown that compassion may enable university students to face, overcome, and bounce back from adversity and generate a greater sense of thriving and meaning in life (Chio et al., 2021). However, the underlying processes are largely unknown. The present study aimed to examine the associations of compassion from others and from the self with psychological distress, flourishing, and meaning in life among university students and explore the mechanisms underlying these associations. We hypothesized that compassion from others would be related positively to self-compassion, which would, in turn, be linked to greater resilience and consequently lower levels of psychological distress and higher levels of flourishing and meaning in life.

## Methods

### Participants

The participants were 536 university undergraduate students in Hong Kong Special Administrative Region, China. Of these, 19.4% were first-year students, 22.8% were second-year students, 24.4% were third-year students, 22.8% were fourth-year students, and 10.6% were fifth-year students. They were affiliated to the faculties of education and human development (23.6%), humanities (26.6%), and liberal arts and social sciences (49.8%). The majority of them were female (82.3%). The mean age was 21.51 years (*SD* = 2.56). Most of them reported having meditated at least once before (73.5%). In particular, 22.6% reported to mediate at least once a week, and 50.9% reported to meditate regularly but less than once a week. On a monthly basis, 38.2% had family incomes of less than HK$20,001 (≈ US$2,568), 21.8% had HK$20,001–HK$30,000 (≈ US$2,568–US$3,852), and 39.9% had more than HK$30,000 (≈ US$3,852). The median monthly family income was HK$20,001–HK$30,000, which was comparable to the median monthly family income in Hong Kong—HK$25,000 (≈ US$3,210) (Census & Statistics Department, 2017).

### Procedures

The participants were recruited through mass emails sent to all undergraduate students and flyers distributed in common areas at a university in Hong Kong Special Administrative Region, China, between November 2020 and February 2021. Individuals interested in joining the study visited a data collection website to read the research purposes and procedures, sign written consent forms, and fill in a set of standardized questionnaires. Each participant received a supermarket coupon of HK$50 (≈ US$6.42) as an incentive. The study was approved by the Human Research Ethics Committee of The Education University of Hong Kong.

### Measures

All the measures in the questionnaires were originally written in English. Their Chinese translated versions were used in this study.

#### Compassion from Others

The participants’ experiences of compassion from others were measured using the 10-item Compassionate Engagement and Action Scale (Gilbert et al., 2017). This scale was developed based on the social mentality theory of compassion (Gilbert et al., 2017). Sample items were “Others reflect on and make sense of my feelings of distress” and “Others think about and come up with helpful ways for me to cope with my distress”. Each item was rated on a 10-point Likert scale, where 1 was “never” and 10 was “always”. The item ratings were averaged, such that higher scores represented higher levels of compassion from others. This scale has been used to indicate university students’ experiences of compassion from others, with good validity and reliability (Steffen et al., 2021). In the present study, its Cronbach’s alpha was 0.95 and McDonald’s omega was 0.95.

#### Self-compassion

The participants’ self-compassion was measured using the 12-item Self-Compassion Scale (Neff, 2003; Raes et al., 2011). This scale was developed based on a social psychological perspective of self-compassion (Neff, 2003; Raes et al., 2011). Sample items were “When I’m going through a very hard time, I give myself the caring and tenderness I need” and “When something painful happens, I try to take a balanced view of the situation”. Each item was rated on a 5-point Likert scale, where 1 was “almost never” and 5 was “almost always”. The item ratings were averaged, such that higher scores represented higher levels of self-compassion. This scale has been used to indicate university students’ self-compassion, with good validity and reliability (Wong & Mak, 2016). In the present study, its Cronbach’s alpha was 0.85 and McDonald’s omega was 0.85.

#### Resilience

The participants’ resilience was measured using the six-item Brief Resilience Scale (Smith et al., 2008). Sample items were “I tend to bounce back quickly after hard times” and “It does not take me long to recover from a stressful event”. Each item was rated on a 5-point Likert scale, where 1 was “strongly disagree” and 5 was “strongly agree”. The item ratings were averaged, such that higher scores represented higher levels of resilience. This scale has been used to indicate university students’ resilience, with good validity and reliability (Lai & Yue, 2014). In the present study, its Cronbach’s alpha was 0.77 and McDonald’s omega was 0.78.

#### Psychological Distress

The participants’ psychological distress was measured using the 21-item Depression Anxiety Stress Scale (Henry & Crawford, 2005). Sample items were “I felt that I had nothing to look forward to” and “I felt I was close to panic”. Each item was rated on a 4-point Likert scale, where 0 was “not at all” and 3 was “most of the time”. The item ratings were averaged, such that higher scores represented higher levels of psychological distress. This scale has been used to indicate university students’ psychological distress, with good validity and reliability (Wang & Du, 2020). In the present study, its Cronbach’s alpha was 0.95 and McDonald’s omega was 0.95.

#### Flourishing

The participants’ flourishing was measured using the eight-item Flourishing Scale (Diener et al., 2010). Sample items were “I am optimistic about my future” and “I am competent and capable in the activities that are important to me”. Each item was rated on a 7-point Likert scale where 1 was “strongly disagree” and 7 was “strongly agree”. The item ratings were averaged, such that higher scores represented higher levels of flourishing. This scale has been used to indicate university students’ flourishing, with good validity and reliability (Xiao et al., 2021). In the present study, its Cronbach’s alpha was 0.90 and McDonald’s omega was 0.90.

#### Meaning in Life

The participants’ meaning in life was measured using the 10-item Meaning in Life Questionnaire (Steger et al., 2006). Sample items were “I understand my life’s meaning” and “I have a good sense of what makes my life meaningful”. Each item was rated on a 7-point Likert scale where 1 was “absolutely untrue” and 7 was “absolutely true”. The item ratings were averaged, such that higher scores represented higher levels of meaning in life. This scale has been used to indicate university students’ meaning in life, with good validity and reliability (Lew et al., 2019). In the present study, its Cronbach’s alpha was 0.80 and McDonald’s omega was 0.79.

#### Background Variables

The participants provided personal background information, including gender, age, education level, major study area, meditation practice, and family income.

##### Data Analyses

Descriptive statistics and correlation tests were performed using SPSS Version 28.0 to examine the descriptives of and inter-correlations among compassion from others, self-compassion, resilience, psychological distress, flourishing, meaning in life, and background variables. Significant background covariates were controlled in the subsequent analyses.

Serial mediation analyses were conducted using PROCESS macro for SPSS Version 4.0 (Model 6; Hayes, 2018) to examine the associations of compassion from others, self-compassion, and resilience with psychological distress, flourishing, and meaning in life. The statistical significance of indirect effects was assessed with the bootstrap method. Based upon 5,000 bootstrapped samples, bias-corrected confidence intervals were generated. The absence of zero from the 95% confidence interval represented a significant indirect effect.

## Results

Table 1 summarizes the results of the descriptive and correlation analyses. Compassion from others, self-compassion, resilience, flourishing, and meaning in life were correlated positively with one another (*p*s < 0.001). All of these variables were correlated negatively with psychological distress (*p*s < 0.001). Of the background variables, male gender was correlated positively with resilience (*p* < 0.05), and family income was correlated positively with flourishing and meaning in life (*p*s < 0.05) and negatively with psychological distress (*p* < 0.01). Based on these findings, gender and family income were included as covariates in the following analyses.

**Table 1:** Descriptive statistics of and correlations among variables

	Range	*M*	*SD*	2	3	4	5	6
1. Compassion from others	1.30–10.00	6.00	1.54	0.31***	0.17***	− 0.23***	0.40***	0.26***
2. Self-compassion	1.58–5.00	3.27	0.58		0.58***	− 0.55***	0.45***	0.31***
3. Resilience	1.17–5.00	3.09	0.61			− 0.50***	0.47***	0.32***
4. Psychological distress	0.00–3.00	0.83	0.60				− 0.53***	− 0.37***
5. Flourishing	1.63–7.00	5.21	0.87					0.68***
6. Meaning in life	2.50–7.00	4.96	0.72					

^***^p < 0.001

**Fig. 1:**
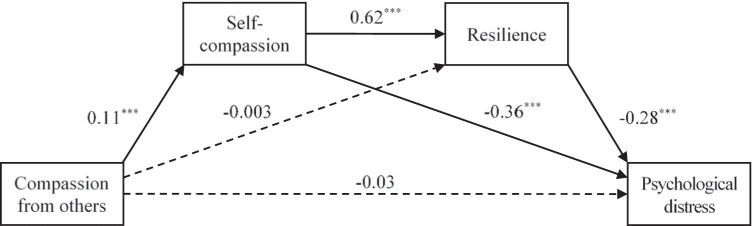
A serial mediation model linking compassion from others to self-compassion, resilience, and psychological distress. Solid lines indicate significant paths, whereas dashed lines indicate non-significant paths. ***p < 0.001

Figure 1 shows the serial mediation model linking compassion from others to self-compassion, resilience, and psychological distress. Compassion from others had significant direct effects on self-compassion (*p* < 0.001), which had significant direct effects on resilience (*p* < 0.001). Both self-compassion (*p* < 0.001) and resilience (*p* < 0.001) had significant direct effects on psychological distress. With the effects of self-compassion and resilience controlled, compassion from others did not have significant direct effects on psychological distress (*p* > 0.05), but had significant indirect effects on psychological distress through self-compassion (indirect effect =  − 0.04, 95% CI = [− 0.06, − 0.03]) and serially through self-compassion and resilience (indirect effect =  − 0.02, 95% CI = [− 0.03, − 0.01]). The total indirect effects of compassion from others on psychological distress were significant (indirect effect =  − 0.06, 95% CI = [− 0.08, − 0.04]). Overall, the model explained 36.9% of the variance in psychological distress.

**Fig. 2:**
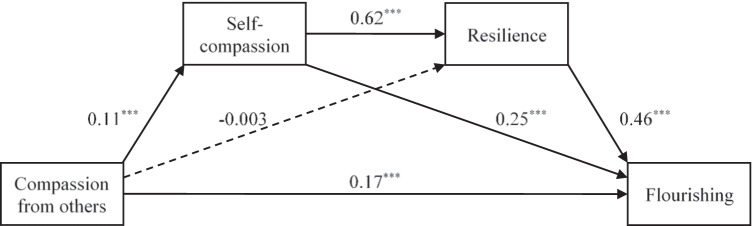
A serial mediation model linking compassion from others to self-compassion, resilience, and flourishing. Solid lines indicate significant paths, whereas dashed lines indicate non-significant paths. ***p < 0.001

Figure 2 shows the serial mediation model linking compassion from others to self-compassion, resilience, and flourishing. Compassion from others had significant direct effects on self-compassion (*p* < 0.001), which, in turn, had significant direct effects on resilience (*p* < 0.001). Both self-compassion (*p* < 0.001) and resilience (*p* < 0.001) had significant direct effects on flourishing. With the effects of self-compassion and resilience controlled, compassion from others had significant direct effects on flourishing (*p* < 0.001). It also had significant indirect effects on flourishing through self-compassion (indirect effect = 0.03, 95% CI = [0.01, 0.05]) and serially through self-compassion and resilience (indirect effect = 0.03, 95% CI = [0.02, 0.05]). The total indirect effects of compassion from others on flourishing were significant (indirect effect = 0.06, 95% CI = [0.03, 0.09]). Overall, the model explained 34.8% of the variance in flourishing.

**Fig. 3:**
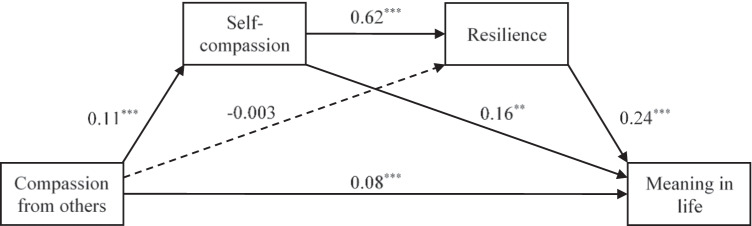
A serial mediation model linking compassion from others to self-compassion, resilience, and meaning in life. Solid lines indicate significant paths, whereas dashed lines indicate non-significant paths. **p < 0.01; ***p < 0.001

Figure 3 shows the serial mediation model linking compassion from others to self-compassion, resilience, and meaning in life. Compassion from others had significant direct effects on self-compassion (*p* < 0.001), and self-compassion had significant direct effects on resilience (*p* < 0.001). Both self-compassion (*p* < 0.01) and resilience (*p* < 0.001) had significant direct effects on meaning in life. With the effects of self-compassion and resilience controlled, compassion from others had significant direct effects on meaning in life (*p* < 0.001) and had significant indirect effects on meaning in life through self-compassion (indirect effect = 0.02, 95% CI = [0.004, 0.04]) and serially through self-compassion and resilience (indirect effect = 0.02, 95% CI = [0.01, 0.03]). The total indirect effects of compassion from others on meaning in life were significant (indirect effect = 0.04, 95% CI = [0.02, 0.05]). Overall, the model explained 15.8% of the variance in meaning in life.

## Discussion

Consistent with our hypotheses, compassion from others was related positively to self-compassion, which was, in turn, linked to greater resilience and consequently lower levels of psychological distress and higher levels of flourishing and meaning in life. These findings suggest that, during difficult times, receiving warmth and care from others may promote self-warmth and self-care, which can enable one to get through, bounce back, and move on from adversities. In turn, such a positive adjustment to difficult circumstances may result in not only less emotional suffering but also a greater sense of thriving and meaning in life.

In line with previous studies showing the contributory role of other-to-self compassion in self-to-self compassion (Gilbert et al., 2011, 2017; Hermanto & Zuroff, 2016), our study found that the experience of receiving and accepting compassion from others may facilitate one to develop a compassionate sense of self. These findings suggest that compassion can ripple or flow out like a contagion: when people feel themselves contextualized in supportive and caring environments, they are also more likely to support and care for themselves (Gilbert, 2014). Importantly, the findings highlight that self-compassion is influenced by the broader social environment and, thus, a social-ecological approach should be used to understand and enhance the development of self-compassion (Gilbert, 2014).

Previous research has demonstrated the positive association of compassion from others with resilience (Van Vliet et al., 2018). Building on this finding, our study showed that the association was mediated, and could be accounted for, by self-compassion. Specifically, our findings reveal that, through receiving compassionate social support, individuals may possess more self-compassionate attitudes as well as resilient mindsets. These findings suggest that compassion from others may have a self-empowering effect that supports individuals to cope with and recover from adversity (Ferreira et al., 2021).

Expanding upon previous studies on the psychological benefits of compassion (Chio et al., 2021; Kirby et al., 2019), our study showed that compassion from others and from the self were associated with less distress and greater flourishing and meaning in life. Specifically, these findings indicate that compassion may facilitate individuals to manage, adapt to, and cope with difficult life events, which may then enable them to transform affective distress to positive affect and generate a sense of meaning in life. These findings suggest that compassion may play an important role in supporting individuals to have positive psychological adjustment amidst challenging or threatening circumstances (Matos et al., 2022).

The present study tested three serial mediation models to elucidate the potential pathways through which compassion from others and self-compassion could facilitate university students to develop the capacity for resilience and lead flourishing and meaningful lives. Importantly, our findings reveal the psychological benefits of compassion from others and from the self for university students. The findings highlight the importance of being open and receptive to love and kindness from others during times of distress (Gilbert et al., 2011). The findings also point to the importance of developing a caring attitude toward oneself when facing hardship or adversity (Neff & McGehee, 2010).

Our models advance the literature by clarifying the links of compassion to well-being and life meaning in university students. As researchers have just started investigating the psychological benefits of compassion for university students (Dvořáková et al., 2019; Noh & Cho, 2020), additional studies are required to establish more comprehensive models explaining how compassion may affect university students’ well-being and life meaning. Specifically, future researchers may examine whether compassion can promote university students’ well-being and life meaning via other mechanisms (e.g., increases in self-reassurance and positive reappraisal and decrease in self-criticism and negative rumination) (Cavalcanti et al., in press; Ewert et al., 2021). Future researchers may also examine whether compassion can moderate and mitigate the adverse effects of stressors on university students’ well-being and life meaning (Matos et al., 2022).

### Limitations and Future Research

Several limitations of the present study should be considered. First, our sample was recruited from only one university and consisted mainly of female students, which might have limited the generalizability of our findings. Future researchers should recruit representative samples of university students with balanced gender ratios in order to further validate our mediation models. Second, our measures of compassion from others and self-compassion were respectively developed by Gilbert et al. (2017) and Neff (2003) based on different theoretical perspectives of compassion, which might have affected the conceptual coherence of our models. Future researchers should assess these variables using measures that were developed based on the same theoretical perspective. Third, our self-report questionnaire measures might have been affected by shared method variance and single reporter bias (Podsakoff et al., 2012). Future researchers should employ various methods (e.g., observations and interviews) and involve multiple informants (e.g., families and friends) in data collection. Fourth, our data were collected during the period of COVID-19, which might have affected the participants’ well-being and life meaning, influencing their responses. Future researchers should reexamine our hypotheses after the pandemic. Fifth, our cross-sectional data did not permit us to draw definitive conclusions about causal relations. Future researchers should conduct longitudinal or intervention studies to further examine if compassion from others and from the self can lead to less distress and greater flourishing and meaning in life.

## Data Availability

The data of this study are available from the corresponding author upon reasonable request.
